# MutL binds to 3′ resected DNA ends and blocks DNA polymerase access

**DOI:** 10.1093/nar/gkac432

**Published:** 2022-06-07

**Authors:** Alessandro Borsellini, Joyce H G Lebbink, Meindert H Lamers

**Affiliations:** Department of Cell and Chemical Biology, Leiden University Medical Center, Leiden, The Netherlands; Department of Molecular Genetics, Oncode Institute, Erasmus MC Cancer Institute, Erasmus University Medical Center, Rotterdam, The Netherlands; Department of Radiation Oncology, Erasmus University Medical Center, Rotterdam, The Netherlands; Department of Cell and Chemical Biology, Leiden University Medical Center, Leiden, The Netherlands

## Abstract

DNA mismatch repair removes mis-incorporated bases after DNA replication and reduces the error rate a 100–1000-fold. After recognition of a mismatch, a large section of up to a thousand nucleotides is removed from the daughter strand followed by re-synthesis. How these opposite activities are coordinated is poorly understood. Here we show that the *Escherichia coli* MutL protein binds to the 3′ end of the resected strand and blocks access of Pol I and Pol III. The cryo-EM structure of an 85-kDa MutL-DNA complex, determined to 3.7 Å resolution, reveals a unique DNA binding mode that positions MutL at the 3′ end of a primer-template, but not at a 5′ resected DNA end or a blunt DNA end. Hence, our work reveals a novel role for MutL in the final stages of mismatch repair by preventing premature DNA synthesis during removal of the mismatched strand.

## INTRODUCTION

DNA mismatch repair is essential for safeguarding the integrity of the genome. It corrects mismatches incorporated during DNA replication, reducing the error rate a 100–1000-fold (1). The absence of mismatch repair leads to increased mutation rates, antibiotic drug resistance in bacteria, and cancer in humans (2,3). The repair process is executed by a series of proteins that together search, find and remove the mis-incorporated nucleotide from the newly synthesized strand. The repair process is initiated when the dimeric MutS protein recognizes a mismatch or a looped-out base among millions of matched base pairs (bp) (4,5). MutS then undergoes a large, ATP-dependent conformational change that results in the recruitment of the second repair protein MutL (6,7). The MutL homologues perform multiple tasks during the repair cascade. In *Escherichia coli*, MutL activates the endonuclease MutH that selectively nicks the newly synthesized strand at hemi-methylated GATC sites which are present on the DNA temporarily after DNA replication (8,9). In other species, including eukaryotes, the MutL homologs themselves contain the endonuclease activity, which is directed to the newly synthesized strand through their interaction with the processivity clamp β or PCNA (10,11). Subsequent to the creation of the single stranded nick, a DNA helicase and exonucleases are recruited to remove the newly synthesized strand between the nick and the mismatch, which may be separated by up to one kilo base pairs (kb) (5,12). During this process, MutL plays an essential role by activating the UvrD helicase to remove a large section of the daughter strand containing the incorrectly incorporated nucleotide (13,14). Subsequent to mismatch removal, a DNA polymerase will resynthesize the removed strand and a DNA ligase will close the gap (5,15). However, it is not well understood how the switch between DNA unwinding and DNA resynthesis is organized. The discovery of a β-clamp binding motif in MutS and MutL (16) and a direct interaction between the replicative DNA polymerase Pol IIIα and MutS and MutL (17), suggest that MutS and MutL recruit the DNA polymerase to the DNA after excision of the newly synthesized strand, but how this is achieved is not clear.

Here, we sought to gain insight into the recruitment of the replication proteins after excision of the newly synthesized strand. Surprisingly, rather than recruiting the replication proteins, we find that MutL inhibits DNA access of Pol I and Pol III to the primer/template junction. This inhibitory action of MutL is dependent on the presence of a mismatch and loading by MutS. We furthermore present the cryo-EM structure of an 85 kDa MutL-DNA complex to 3.7 Å resolution, which reveals how MutL specifically binds to a 3′ resected primer/template junction, the substrate for DNA polymerases, but not to a 5′ resected DNA end or blunt-ended DNA. Mutation of two conserved residues that interact with the 3′ resected end results in loss of end-blocking and a mutation phenotype *in vivo*.

Thus, our work provides new insights into the final stages of the mismatch repair process and reveals a role for MutL in preventing premature DNA synthesis during removal of the mismatched strand.

## MATERIALS AND METHODS

All chemicals were purchased from Sigma Aldrich, unless indicated otherwise. All chromatography columns were purchased from Cytiva.

### Protein expression and purification

Subunits of the Pol III Holoenzyme (α, β, ϵ, τ, δ, δ’), Pol I Klenow fragment and MutS were expressed and purified as described before (18–20). MutL^WT^, MutL^H270A^, MutL^H319A^ and MutL^H270A+H319A^ were transformed into *E. coli* BL21(DE3) T7 express cells, overexpressed for 2 h at 30 °C and purified as follows. Briefly, cells were lysed by sonication in 25 mM HEPES pH 7.5, 500 mM NaCl, 10 mM imidazole, 5 mM MgCl_2_, 2 mM DTT. The supernatant was injected onto HisTrap HP 5ml and eluted with a gradient of the same buffer complemented with 500 mM imidazole. Pooled fractions were incubated with 10% (g/ml) ammonium sulphate for 10 minutes and later centrifuged. The supernatant was loaded onto HiTrap Phenyl column 5 ml and eluted with 25 mM HEPES pH 7.5, 5 mM MgCl_2_, 2 mM DTT. Pooled fractions were injected onto HiTrap Heparin column 5ml and eluted with a gradient of 25 mM HEPES pH 7.5, 1 M NaCl, 5 mM MgCl_2_, 2 mM DTT. Pooled fractions were concentrated and injected into a gel filtration column Superdex 200 16/600 equilibrated in 25 mM HEPES pH 7.5, 150 mM NaCl, 5 mM MgCl_2_, 2 mM DTT, 5% glycerol. All proteins yielded similar amounts of purified protein and were snap-frozen in liquid nitrogen right after purification and stored at −80°C.

### ΦX174 primer extension assays

Protein activity was tested using single stranded ΦX174 phage DNA (New England Biolabs, Hitchin, United Kingdom), primed with a 5′ fluorescein labelled primer (sequence: 5′-FAM-TAGAGTCAATAGCAAGGCCACGACGCAATGGAGAAAGACGGAGAG-3′, or 5′-FAM-TAGAGTCAATAGCAAGGCCACGgCGCAATGGAGAAAGACGGAGAG-3′, where lower case ‘g’ indicates the position of the GT mismatch). Reactions were performed in 20 mM Tris pH 7.5, 2 mM DTT, 150 mM potassium glutamate, 8 mM magnesium chloride and 0.05 mg/ml BSA. Each reaction contained 5 nM primed ΦX174 phage DNA, 1 μM SSB, 20 nM β clamp, 2 nM τ clamp loader complex (τ_3_δ_1_δ’_1_), 4 nM polymerase (Pol III-core), 20 nM Pol I Klenow fragment and 40 nM MutS and MutL. Reactions were quenched at different time points between 0 and 15 min with stop buffer (75 mM EDTA and 0.6% (W/V) SDS) and separated on an alkaline agarose gel (0.8% agarose, 30 mM NaOH, 2 mM EDTA) for 15 hr at 15 V. Gels were scanned at 488 nm using an Amersham Typhoon (GE Healthcare). Preparation of partial dsDNA ΦX174 was performed with a primer extension reaction in presence of Pol I Klenow fragment. The reaction started from the 5′-fluorescein labelled mismatched primer and was quenched with stop buffer after 60 s before the full circular dsDNA product could be reached. To do the reaction, we used 10 molar excess of Pol I Klenow. This way every ΦX174 DNA molecule was simultaneously a substrate for DNA synthesis and the length of the dsDNA stretch was homogeneous among the different DNA molecules. Next, the DNA was purified from the proteins using a Monarch PCR & DNA Cleanup Kit. All primer extension assays were performed two or three times independently.

### Short DNA substrate primer extension assays

Primer extension assays were performed using the following primers: 5′-CGCTCACTGGCCGTCGTTTTACAACGTCGTCTAGATTCGTGAAGTGACTGGGAAAACCCTGGCGTTACC-3′ and 5′- Cy3-GGTAACGCCAGGGTTTTCCCAGTC-3′. Assays were performed in 20 mM HEPES pH 7.5, 2 mM DTT, 5 mM MgCl_2_, 50 mM NaCl and 0.05 mg/ml BSA. Reactions were performed at 22 °C with 300 nM polymerase III α subunit, 1.5 μM MutL and 100 nM of DNA substrate. Primer extensions were carried out for 90 seconds in the presence of 100 μM dNTP’s (unless stated otherwise). Reactions were stopped in 35 mM EDTA and 65% formamide and separated on a denaturing 20% acrylamide/bis-acrylamide (19:1) gel with 7.5 M urea in 1× TBE for 80 min at 30 W. The gel was imaged with a Typhoon Imager (GE Healthcare).

### Fluorescence anisotropy DNA binding assays

Fluorescence anisotropy measurements were performed in a Clariostar plate reader. The measurements were taken at an excitation wavelength of 460 nm and an emission wavelength of 515 nm. Dilutions of MutL ranging from ∼30 nM to 4 μM were incubated with three different substrates: 3′ resected primer/template junction, a 5′ resected primer/template junction, and a blunt-ended DNA. In all three substrates the DNA duplex was 15 bp, while the overhang was 16 bp. The sequences of primers used for the assays were: template duplex 5′-AAGTGTCTGTCGTTTT-3′; template for 5′ and 3′ resected primer/template junction 5′-AAGTGTCTGTCGTTTTAAGGACGAAGGACTC-3′; primer for duplex and 5′ resected DNA substrate 5′-AAAACGACAGACACTT-3′-FAM; primer for 3′ resected DNA substrate FAM-5′-GAGTCCTTCGTCCTT-3′. The final DNA concentration used for assays was 5 nM. All binding reactions were carried out in buffer containing 20 mM HEPES pH 7.5, 150 mM NaCl, 5 mM MgCl_2_, 0.08% Tween 20, 20% glycerol and 2 mM DTT. Reactions were performed in presence of 3 mM ATP, 3 mM AMPPNP or without nucleotides. The anisotropy of each sample was measured at 25°C in Corning 384-well Black Round Bottom well plates. Three measurements were collected and averaged for each point of the binding isotherm. The dissociation constant (*K*_d_) values and standard error of the mean were calculated in Graphpad Prism, using all data points from three independent experiments (24 data point total).

### Cryo-EM sample preparation and imaging

Purified MutL was diluted to 4 μM in 20 mM Tris pH 8.5, NaCl 150 mM, 5 mM MgCl_2_, 2 mM DTT, 0.01% Tween 20. The diluted protein was incubated for 5 minu with 3 mM AMPPNP and 20 μM DNA with a 3′ resected end (template: 5′-AAACAGGCTTAGGCTGGA-GGATCAGCTTAGCTTAGAGTCATC-3′, primer: 5′-GATGACTCTAAGCTAAGCTGA-3′). Three μl of sample at 4 μM were adsorbed onto glow-discharged copper Quantifoil R2/1 holey carbon grids (Quantifoil). Grids were glow discharged 45 s at 25 mA using an EMITECH K950 apparatus. Grids were blotted for 1 s at ∼80% humidity at 4°C and flash frozen in liquid ethane. Cryo-EM grids were prepared using a Leica EM GP plunge freezer. The grids were loaded into a Titan Krios (FEI) electron microscope operating at 300 kV with a Gatan K3 detector. The slit width of the energy filter was set to 20 eV. Images were recorded with EPU software (https://www.fei.com/software/epu-automated-single-particles-software-for-life-sciences/) in counting mode. Dose, magnification and effective pixel size are detailed in Table 1.

**Table 1.** Cryo-EM data collection, refinement and validation statistics

**Data collection and processing**		**Model comparison**	
Magnification	×105 000	Nonhydrogen atoms	5990
Voltage (kV)	300	Protein residues	665
Electron exposure e^–^/Å^2^	54	*B* factors (Å^2^)	
Defocus range (μm)	0.8 to 2.0	Protein	28–633
Pixel size (Å)	0.84	r.m.s. deviations	
Symmetry imposed	C1	Bond lengths (Å^2^)	0.0060
Initial particle images (no)	539000	Bond angles (°)	1.3192
Final particle images (no)	149000	**Validation**	
Map resolution (Å)	3.6	MolProbity score	1.49
FSC threshold	1.43	Clashscore	5.24
Map resolution range (Å)	3.6 to >5.5	Poor rotamers (%)	0.36
**Refinement**		Ramachandran plot	
Initial model used	1NHI	Favoured (%)	96.66
Model resolution (Å)	3.7	Allowed (%)	3.44
FSC threshold	0.143	Disallowed (%)	0.0
Map sharpening *B* factor (Å^2^)	70		

### Cryo-EM image processing

All image processing was performed using Relion 3.1 (21). The images were drift corrected using Relion's own (CPU-based) implementation of the UCSF motioncor2 program, and defocus was estimated using CTFFIND4.1 (22). LoG-based auto-picking was performed on a subset of micrographs, and picked particles were 2D classified. Selected classes from the 2D classification were used as references to autopick particles from the full data sets. After three rounds of 2D classification, classes with different orientations were selected for initial model generation in Relion. The initial model was used as reference for 3D classification into different classes. The selected classes from 3D classification were subjected to 3D auto refinement followed by Bayesian polishing. Polished particles were used for 3D classification. Selected particles were subjected to different rounds of CTF refinement plus a final round of Bayesian polishing. Finally, we performed particle subtraction followed by 3D classification without image alignment using a regularization parameter equal to 20. Selection of particles was used for 3D auto-refine job and final map was post-processed to correct for modulation transfer function of the detector and sharpened by applying a negative B factor, manually set to −70. A soft mask was applied during post processing to generate FSC curves to yield a map of average resolution of 3.7 Å. The final RELION postprocessed map was used to generate improved-resolution EM maps using the SuperEM method (23), which aided in model building and refinement.

Model building was performed using Coot (24), REFMAC5 (25), the CCPEM-suite (26) and Phenix (27). Details on model refinement and validation are in Table 1. In brief, model building started by rigid-body fitting of the known N-terminal *E. coli* MutL crystal structure (PDB 1NHI) (28) into experimental density map using Coot. The DNA molecule was generated and rigid body fitted into experimental density map using coot. Next, we carried out one round of refinement in Refmac5 using jelly-body restraints, and the model was further adjusted in Coot. A final refinement round and validation of the model and data were carried out using Refmac5 with proSmart (29) restraints and MolProbity (30) within the CCPEM suite respectively.

### Biolayer interferometry dna binding assays

Biolayer interferometry measurements were performed using an Octet-red instrument RED96 (ForteBio). All binding studies were performed with Streptavidin (SA) biosensors (ForteBio) conjugated to the biotinylated DNA substrate. The DNA substrate was prepared as follows: monovalent streptavidin and a 5′ biotinylated primer were mixed in a 1:1 ratio at a concentration of 20 μM and purified via analytical gel filtration using a Superdex 200 increase (3.2/30) column equilibrated in Tris 10 mM pH 8.0, NaCl 50 mM, EDTA 1mM. Fractions containing monovalent streptavidin bound to the biotinylated primer were mixed with the 5′ biotinylated DNA template to create the final DNA substrate. All the experiments were performed in the following buffer: HEPES 25 mM pH 7.5, NaCl 150 mM, MgCl_2_ 5mM, DTT 2mM, BSA 0.5 mg/ml, Tween 20 0.01%, 2 mM ATP. The DNA substrate was added in the loading step at 100 nM, until the threshold value of 0.36 nm was reached. Binding of MutL^WT^, MutL^H270A^, MutL^H319A^ and MutL^H270A+H319A^ was performed at concentrations ranging from 18.75 to 150 nM in presence or absence of 100 nM MutS. Experiments were performed two times independently, with 8 concentrations each.

### Rifampicin fluctuation assays

The number of resistant colonies of MutL mutants were determined using a rifampicin fluctuation assay as described by (31). In brief, a MutL deficient *E. coli* strain (32) was transformed with a plasmid expressing wild type MutL, MutL^H270A^, MutL^H319A^ or MutL^H270A+H319A^, and cells where plated on LB/agar plates with 100 μg/ml ampicillin and 30 μg/ml kanamycin. After overnight incubation at 37°C, single colonies were picked and grown in 10 ml LB with antibiotics to OD600 ∼1.0. Next, 100 μl of cells at OD_600_ ∼0.3 were plated on LB plates with ampicillin, kanamycin and 0.1 mg/ml rifampicin. Plates were incubated overnight at 37°C and rifampicin resistant colonies were counted. Mutation rates (here defined as: probability of a mutation per cell per generation) and 95% confidence intervals were determined using the Fluctuation AnaLysis CalculatOR (https://lianglab.brocku.ca/FALCOR/) using the MSS-MLE method. Mutation rates values are expressed as in ‘number ± 95% CI × 10^*X*^*’*. For both WT and mutants, the number of colonies from nine independent experiments were counted.

## RESULTS

### MutL is a mismatch and MutS-dependent inhibitor of DNA synthesis

**Figure 1.**
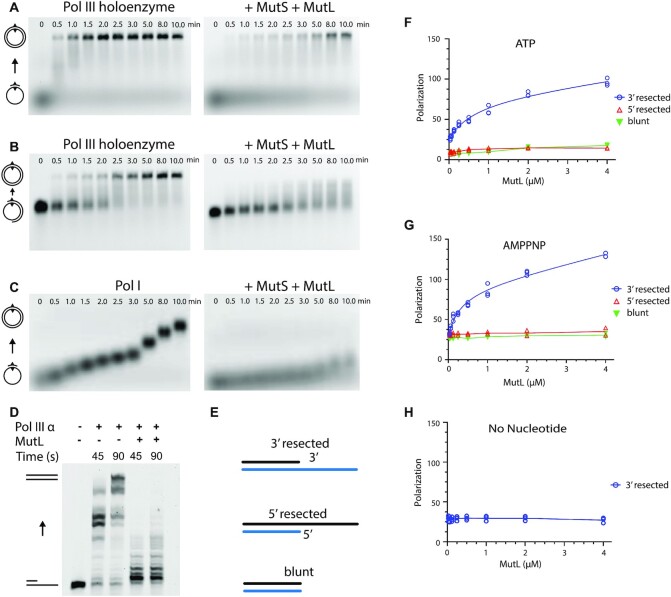
MutL blocks DNA synthesis by binding to 3′ resected ends in a mismatch-dependent manner. (**A**) Primer extension by the *E. coli* Pol III holoenzyme in the absence (left) and presence of MutS and MutL (right) on single stranded ΦX174 phage DNA (5386 bases) primed with a 45-nucleotide primer with a mismatch located at position 23. Numbers above the gel mark time in minutes. Controls without MutS, MutL, or a mismatch are shown in Supplementary Figure S1A and S1B. All experiments were repeated two or three times independently (**B**) Similar extension using a ∼1000 nucleotide primer with the mismatch located at position 23, in absence (left) and presence (right) of MutS and MutL. (**C**) Primer extension by *E. coli* Pol I DNA polymerase in the absence (left) and presence (right) of MutS and MutL. (**D**) Primer extension assay on a linear DNA substrate with Pol III α in presence and in absence of MutL. (**E**) DNA substrates used in the DNA binding studies: a 3′ resected primer/template junction, a 5′ resected primer/template junction, and a blunt-ended DNA substrate. (**F**) Binding of MutL to the DNA substrates in the presence of ATP measured by fluorescence anisotropy. (**G**) Binding of MutL to the DNA substrates in the presence of AMPPNP. (**H**) Binding of MutL to a 3′ resected DNA substrate in the absence of nucleotides.

In order to reveal how the interchange between DNA mismatch repair and DNA synthesis is regulated, we performed primer extension assays on a single stranded ΦX174 phage DNA annealed with a 45-nucleotide primer containing a mismatch in the middle of the sequence (Figure 1A). In the presence of the *E. coli* Pol III holoenzyme subunits α, β, ϵ and the τ-complex (containing the subunits δ_1_τ_3_δ‘_1_) we observe robust DNA synthesis. Surprisingly, when we add MutS and MutL, DNA synthesis is strongly suppressed (Figure 1A right panel). This effect is not observed in the presence of MutS or MutL alone, nor in the absence of a mismatch (Supplementary Figure S1A and S1B). The inhibition of DNA synthesis is also observed when we use DNA substrate with a long primer where the mismatch is located ∼1000 bp away from the 3′ end of the primer (Figure 1B), consistent with the observation that MutS and MutL can move away from the mismatch and slide along the DNA over long distances (33,34). Furthermore, the mismatch repair dependent inhibition is not unique to Pol III alone, as a strong reduction of DNA synthesis is also observed with DNA Pol I (Figure 1C). This suggests that inhibition is not achieved through specific interaction with one of the replication proteins, but more likely through a nonspecific blocking of the 3′ end of a primer/template junction.

### MutL selectively binds 3′ resected DNA ends with high affinity

As Pol I and Pol III both bind to a primer-template junction with a 3′ resected end, we wondered if the inhibitory effect of the mismatch proteins is achieved through direct competition by binding to the 3′ DNA end. Furthermore, as the inhibitory effect is not observed for MutS alone, we postulated that it may be MutL that specifically binds the 3′ resected end. To determine if this is indeed the case, we used fluorescence anisotropy to investigate the binding properties of MutL to a partial duplex DNA with either a 3′ resected end, a 5′ resected end, or a blunt end. (Figure 1E). We performed our assays at physiological salt concentration (150 mM NaCl), as MutL binds non-specifically to DNA at low salt concentrations (50 mM) (14,35,36). Binding was furthermore assayed in presence or absence of 3 mM ATP or AMPPNP. We find that in the presence of ATP MutL specifically binds to a DNA substrate with a 3′ resected end (*K*_d_ = 650 ± 320 nM), but not to 5′ resected end or blunt ended DNA (Figure 1F). Replacing ATP with the non-hydrolysable ATP analogue AMPPNP increases the affinity ∼ 2-fold (*K*_d_ = 290 ± 120 nM) (Figure 1G), whereas no binding is observed in the absence of ATP (Figure 1H). As ATP induces dimerization of the N-terminal ATPase domains of MutL (37), it suggests that the 3′ end binding may be performed by the dimeric form of the N-terminal MutL ATPase domains. This also explains the increased affinity in presence of AMPPNP because this non-hydrolysable ATP analogue keeps the MutL longer in the dimeric form than ATP (37).

The direct binding of MutL to a 3′ resected DNA end seemingly contrasts with the requirement for a mismatch and MutS in the primer extension assays described above. However, the circular DNA used in the primer extension assay will be inaccessible to a closed MutL dimer, and therefore require MutS to load MutL onto the DNA, while the DNA substrates used in the fluorescence anisotropy studies are open-ended, onto which the ATP-induced closed dimer of MutL can thread itself. To verify this, we have performed a primer extension assay on a short linear DNA substrate of 69 bp using Pol III α and MutL^WT^ in the absence of MutS (Figure 1D). We indeed see inhibition of DNA synthesis when MutL is present, confirming that on a short open ended DNA substrate, MutL can inhibit DNA polymerases on its own, but that on a circular DNA it needs MutS and a mismatch to be loaded onto the DNA.

### Cryo-EM structure of MutL reveals mechanism of 3′ resected end recognition

**Figure 2.**
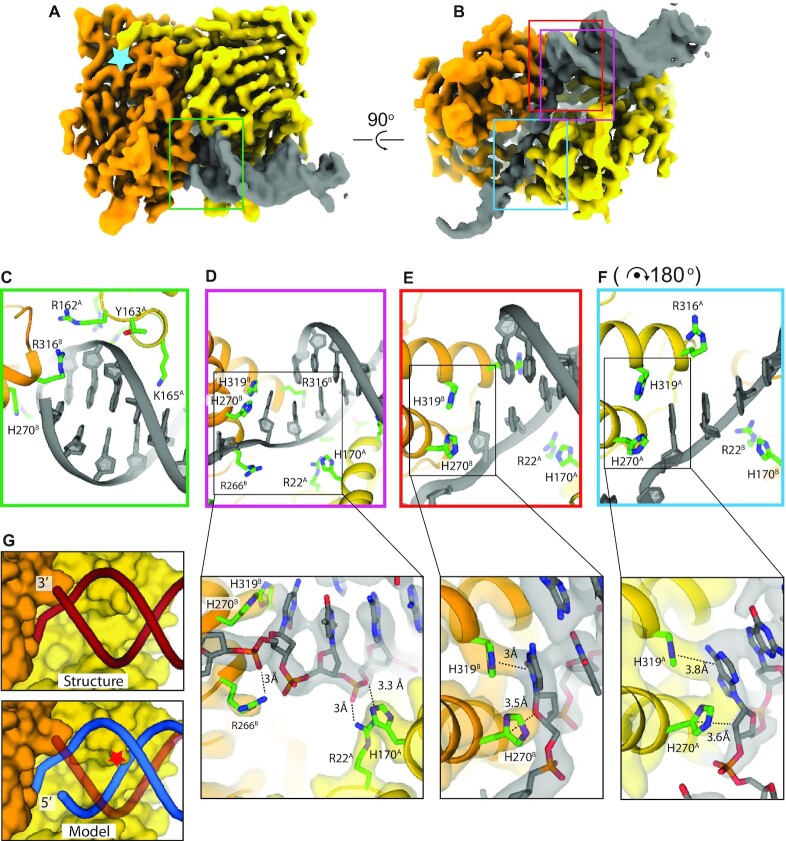
Structure of MutL bound to a 3′ resected DNA end. (**A**) front view and (**B**) bottom view of the cryo-EM map of the MutL ATPase domain dimer bound to a 3′ resected primer-template junction. Monomer A is coloured in yellow, monomer B in orange. Light blue star represents the position of the ATP binding pocket. Coloured squares mark the areas of the close ups shown in (C)–(F). (**C**) Backbone binding of the double stranded section. (**D**) Binding of the ds/ssDNA junction with additional close up of the interaction between R22, H170 and R266 with the phosphates of the DNA. (**E**) ds/ssDNA binding with additional close up on the interaction between H270 and H319 and the first unpaired nucleotide of the continuous strand. (**F**) Binding to the single stranded overhang that shows a similar interaction between histidines H270 and H319 and an unpaired base. For easy comparison, the panel is rotated 180° with respect to panel (E). Close ups in figure (D), (E) and (F) display the experimental cryo-EM map coloured relatively to the chains. (**G**) Comparison between the structure (top) and a predicted model of a 5′ resected DNA substrate after superposition of the continuous strand (bottom). Red star indicates the clash of the backbone of the 5′ resected strand with K165 and the helix it is located on.

In order to elucidate how MutL preferentially binds to 3′ resected DNA ends we determined the cryo-EM structure of MutL bound to 21 bp double stranded DNA substrate with a 21-nucleotide single stranded overhang in the presence of the non-hydrolysable ATP analogue AMPPNP to 3.7 Å resolution (Figure 2A and B and Supplementary Figure S2). Although we used full length MutL during sample preparation, in our structure we only see the N-terminal ATPase dimer and 13 bp of dsDNA plus 8 nucleotides of the single stranded overhang, resulting in a protein-DNA complex with a combined molecular weight of ∼85 kDa. The C-terminal domain of MutL is not visible in the 2D classes or final 3D map, most likely due to the ∼100 amino acid unstructured linker that connects the N-terminal and C-terminal domains. Two N-terminal ATPase domains of MutL (MutL^LN40^) form the canonical dimer confirmation similar to the crystal structure of AMPPNP-bound MutL^LN40^ (37) with both monomers containing an AMPPNP molecule bound in the active site (Supplementary Figure S2E). The only deviation from the crystal structure are two helices that straddle the single stranded DNA overhang and move inward in the DNA bound structure (Supplementary Figure S2G). The symmetry of the MutL^LN40^ dimer is broken by the DNA, with the double stranded section positioned on one end of the dimer, and the single stranded section on the opposite end (Figure 2A, B).

The double stranded section of DNA substrate is predominantly bound by monomer A, shown on the right of Figure 2A-B, which interacts with the DNA backbone through residues 161–165 (Figure 2C), and residues R22 and H170 (Figure 2D). The single stranded overhang passes through a narrow cavity between the two MutL monomers and interacts with residues from both monomer A and B in a sequence independent manner (Figure 2B and F). Several of these residues, R162, R266 and R316, have previously been shown to be important for DNA binding (9,31) or to come close to the DNA in the structure of MutS-DNA-MutL^LN40^ (R22, R62, H170, R266) (38). Two additional mutants that have been described to affect DNA binding, K159E and R177E, (9) are distant from the DNA in our structure and are more likely to be involved in folding and/or dimerization of the protein. The specific recognition of the 3′ end of the dsDNA is performed by monomer B that thus positions the MutL dimer at the same DNA end that is the substrate for DNA polymerases. To do so, it interacts with the last base of the primer strand through a helix (residues 315–330) that positions R316 in the minor groove of the dsDNA (Figure 2C). In addition, two histidines interact with the first unpaired nucleotide: H319 stacks onto the base, while H270 stacks onto the ribose of the same nucleotide (Figure 2E). The same stacking interaction is also observed in the opposite monomer, where H319 and H270 interact with the ssDNA (Figure 2F).

The structure furthermore explains why MutL selectively binds to 3′ resected DNA end. Positioning of a dsDNA substrate with a 5′ resected end results in a clash with monomer A, where K165 that normally protrudes into the major groove, now runs into one strand of the DNA (Figure 2C, blue line). A blunt-ended DNA substrate is also not compatible with binding to MutL, as it lacks the single stranded DNA strand that provides much of the interaction between MutL and the DNA.

### H270 and H319 are essential for polymerase inhibition

**Figure 3.**
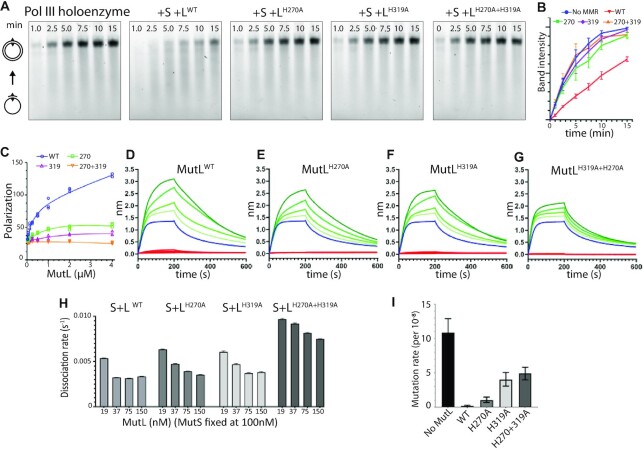
Residues H270 and H319 are required for polymerase blocking and mismatch repair. (**A**) Primer extension assay using wild type (WT) and mutant versions of MutL. (**B**) Band intensities of the top band in primer extension assays of panel A. (**C**) Binding of wild type and mutant MutL to a 3′ resected DNA substrate in presence of 1 mM AMPPNP using fluorescence anisotropy. **(D–G**) Biolayer interferometry measurement of MutS dependent loading of MutL onto an end-blocked DNA substrate with a single mismatch using wild type and mutant MutL. Red lines indicate MutL binding in absence of MutS. Blue line indicates binding by MutS alone. Green lines indicate binding of MutS and increasing concentrations of MutL. Concentrations used are 100 nM MutS and 19–150 nM MutL. (**H**) Dissociation rates extrapolated from BLI measurements. (**I**) Mutation rate of a MutL-deficient *E. coli* strain complemented with wild type MutL, MutL^H270A^, MutL^H319A^ or MutL^H270A+H319A^. Bars represent 95% confidence intervals.

The structure of MutL on a primer/template junction described above indicates a crucial role for the two histidines H270 and H319 in binding of the 3′ resected end. To further assess the role of these two histidines we mutated each residue into an alanine and determined their capability of inhibiting DNA synthesis. Indeed, a single point mutation of either histidine (MutL^H270A^ and MutL^H319A^) as well as the double mutant (MutL^H270A+H319A^) abolish the inhibition of polymerase activity (Figure 3A-B). Similarly, both single mutants show a marked decrease in binding to a 3′ resected DNA substrate, while the double mutant shows a complete loss of DNA binding (Figure 3C and Table 2). Remarkably, the H270A mutant appears to have a slightly less pronounced impact on blocking of DNA synthesis and DNA binding than the H319A mutation. All three mutants yielded similar amounts of purified protein, showed thermal denaturation profiles similar to wild type MutL and all retained AMPPNP induced dimerization (Supplementary Figure S3 A-B), excluding the possibility that the mutations resulted in unfolding or destabilization of the protein.

**Table 2.** Dissociation constants (*K*_d_) of MutL WT and mutant versions for 3′ resected DNA ends

	AMPPNP	ATP
MutL^WT^	0.29 ± 0.12 μM	0.64 ± 0.32 μM
MutL^H270A^	1.59 ± 0.23 μM	*n.d*.
MutL^H319A^	4.51 ± 1.18 μM	*n.d*.
MutL^H270A + H319A^	*n.d*.	*n.d*.

To also exclude the possibility that the loss of inhibition is caused by a defect in loading of MutL onto dsDNA by MutS, we measured the formation of MutS–MutL complexes using wild type and the mutant versions of MutL. For this, we used bio-layer interferometry to measure the binding of MutL on a 70 bp, end-blocked DNA substrate containing a single mismatch. In the absence of MutS, no binding of MutL to the DNA is observed in agreement with previous studies (6,39) (Figure 3C to F). In presence of MutS however, we see a build-up of the signal with increasing concentrations of MutL, due to the formation of stable MutS–MutL complexes on the DNA (Figure 3D–G). The binding profiles of the single mutants are similar to wild type MutL, which is also reflected in their dissociation rates that slow down with increasing concentrations of MutL due to the formation of the stable MutS–MutL complex (Figure 3H). The double mutant however shows a reduced build-up of the signal, possibly due to the loss of two positively charged residues in the central DNA binding cavity of the MutL dimer. Taken together, these results show that H270 and H319 play an essential role in 3′ resected end binding, and that loss of either residue results in a loss of polymerase inhibition.

### H270 and H319 are important for mismatch repair in vivo

To assess the role of 3′ resected end-binding of MutL for mismatch repair as a whole, we also examined the impact of the mutations H270A, H319A and the double mutant in DNA mismatch repair *in vivo*. For this we used a MutL deficient strain (32) which was transformed with a plasmid expressing either wild-type MutL, MutL^H270A^, MutL^H319A^ or MutL^H270A+319A^. Subsequent to transformation, cells were plated onto rifampicin containing plates and resistant colonies counted the next day. In absence of MutL the mutation rate (probability of a mutation per cell per generation) is 1.1 ± 0.2 × 10^–7^, while transformation of wild type MutL reduces the mutation rate to 1.2 ± 0.1 × 10^–9^. In contrast, transformation with MutL^H270A^, MutL^H319A^ and MutL^H270A+H319A^ generates mutation rates of 1.0 ± 0.5 × 10^–8^, 4.0 ± 1.0 × 10^–8^ and 4.9 ± 0.9 × 10^–8^, respectively. These partial mutator phenotypes likely reflect the effect of the mutations on MutL binding to the resected 3′ nick, while 5′-nick directed repair is not affected. Interestingly, MutL^H319A^ has a more pronounced mutator phenotype compared to MutL^H270A^, which correlates with the primer extension assays and DNA end-binding data showing a more enhanced effect of the H319A mutation compared to the H270A mutation (Figure 3A and C). This correlation suggests that the blocking of 3′ resected ends by MutL is also important in vivo.

## DISCUSSION

During mismatch repair, a large section of up to a 1000 nucleotides of the DNA daughter strand is removed (40), after which a DNA polymerase will resynthesize the resected DNA strand, yet how the replication proteins are recruited is not known. The discovery of a β-clamp binding motif in MutS and MutL (16) and direct interaction between the replicative DNA polymerase Pol IIIα and MutS and MutL (41,17), suggested that MutS and MutL may directly recruit the replisomal proteins. However, examination of the two β-binding motifs in MutS revealed that mutation of the N-terminal motif results in an unstable protein which is the probable cause for the mismatch repair deficient phenotype, while mutation of the C-terminal β-binding motif does not result in mutator phenotype (17). Furthermore, the interaction between the β-clamp and MutL is strictly required in *Bacillus subtilis* where the β-clamp greatly stimulates the endonuclease activity of MutL, but less so in *E. coli* where the endonuclease activity resides in MutH (42). This therefore suggest that the mismatch repair proteins may not be involved in actively recruiting the DNA replication proteins for the resynthesis step.

**Figure 4.**
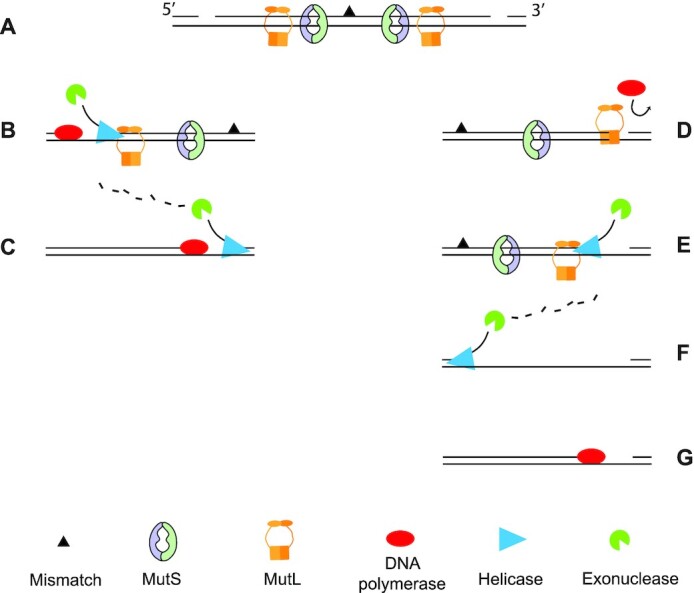
Strand excision and resynthesis during DNA mismatch repair. (**A**) Mismatch repair is bidirectional with MutS and MutL molecules loaded both 5′ and 3′ to the mismatch. Consequently, strand incision can occur on both sides of the mismatch. (**B**) When strand incision is 5′ of the mismatch then the helicase and polymerase work in the same direction. The helicase processivity is enhanced by the C-terminal domain of MutL, while the polymerase follows the excision. (**C**) The displaced strand is removed by an exonuclease until shortly after the mismatch where without the aid of MutL, the helicase lacks processivity and terminates strand removal. (**D**) When the incision occurs 3′ to the mismatch, the polymerase moves opposite to the direction of the helicase and is therefore blocked by MutL that is bound to the 3′ resected end of the newly synthesised strand. (**E**) UvrD associates with the C-terminal domain of MutL that increases the processivity of the helicase. (**F**) An exonuclease removes the displaced strand up to shortly after the mismatch, followed by (**G**) resynthesis of the excised strand.

Here, we propose a novel function of *E. coli* MutL, which is to block access of DNA polymerases to 3′ resected ends, a function that is likely required when strand removal is initiated downstream from the mismatch. Strand discrimination and the subsequent daughter strand removal during mismatch repair is bidirectional as it can initiate both 3′ or 5′ from the mismatched base (5,12) (Figure 4A). When the incision of the daughter strand takes place 5′ of the mismatched base (i.e. upstream), the removal of the daughter strand as well as the resynthesis step take place in the same direction (Figure 4B). Therefore, DNA synthesis can initiate while the strand containing the mismatch is still being removed and digested by an exonuclease (Figure 4C).

In contrast, when the incision of the daughter strand occurs 3′ to the mismatched base (i.e. downstream), the helicase and the polymerase compete for the 3′-end. The action of the polymerase on this end is opposite to that of the helicase and therefore needs to be prevented until after mismatch removal. Our work indicates that MutL could play a pivotal role in this regulation by blocking the access of DNA polymerases to the 3′ end of a resected DNA strand in a mismatch and MutS-dependent manner (Figure 4D). Therefore, as long as the mismatch remains in the DNA, more MutL molecules will be loaded onto the DNA by MutS and ensure a continuous occupation of the 3′ resected end by MutL. Importantly, MutL is also known to enhance the activity of the helicase UvrD during strand excision, through the interaction with the C-terminal part of MutL (43,35), which is not involved in 3′ end binding (Figure 4E). Hence, it is possible that the interaction between UvrD and the C-terminal domain of MutL may trigger the release of the N-terminal domains from the 3′ resected end and initiate strand removal. Then, once the helicase/exonuclease has removed the mismatch (Figure 4F) and no more loading of MutL occurs, DNA polymerase gains access to the 3′-resected end and can re-synthesize the excised strand (Figure 4G).

Interestingly, also in the eukaryotic system there is asymmetry in the excision step of the mismatched strand. In the eukaryotic MMR system however, this is achieved in a different manner (for a recent review, see (44). Here, excision from the 5′ end of a nick in the daughter strand can take place with only MutSα, RPA and EXO1 (45,15). In contrast, repair directed by a 3′ nick also requires an endonuclease proficient MutLα, PCNA and RFC indicating that additional incisions are required, located 5′ of the mismatch to allow excision by the 5′-3′ activity of EXOI (46,47). Alternatively, 5′- and 3′-nick directed repair is carried out by MutSα, RPA, endonuclease-proficient MutLα and Pol δ through synthesis-driven strand displacement (48). Hence, the eukaryotic system appears to favour resection from the 5′ end over the 3′ end, thereby bypassing the problem of the opposite activities of a DNA polymerase and 3′-5′ excision and the need for polymerase inhibition by MutL.

Our work shows that binding of MutL to a 3′-resected DNA end is important for correct timing of DNA resynthesis during mismatch repair. Because MutL interacts with multiple other mismatch repair proteins (MutS, MutH, UvrD) as well as with multiple DNA reaction intermediates, it is possible that resected end-binding is also relevant at other reaction steps along the MMR pathway. This is currently being investigated in our laboratories. In summary, the work described in this study provides new insights into the final stages of the *E. coli* DNA mismatch repair and how the exchange between strand resection and resynthesis is regulated. Instead of a recruitment of a DNA polymerase to the resected strand, we propose that the MutL protein acts as a ‘traffic cop’ on DNA that blocks access to the DNA polymerases at 3′ resected end while stimulating the action of the UvrD helicase to remove the mismatched DNA strand, until the mismatch is removed.

## DATA AVAILABILITY

Atomic coordinates and structure factors for the reported crystal structures have been deposited with the Protein Data bank under accession number PDB 7P8V and EMDB EMD-13255.

## Supplementary Material

gkac432_Supplemental_Files
