# Supplementary material for: MutL binds to 3′ resected DNA ends and blocks DNA polymerase access
Source: Nucleic Acids Res. 2022 Jun 7;50(11):6224–34. doi: 10.1093/nar/gkac432 (PMC9226502; doi:10.1093/nar/gkac432)
Supplement: gkac432_Supplemental_Files [file gkac432_supplemental_files.zip › SupFig_S1_DNA_synthesis.pdf]

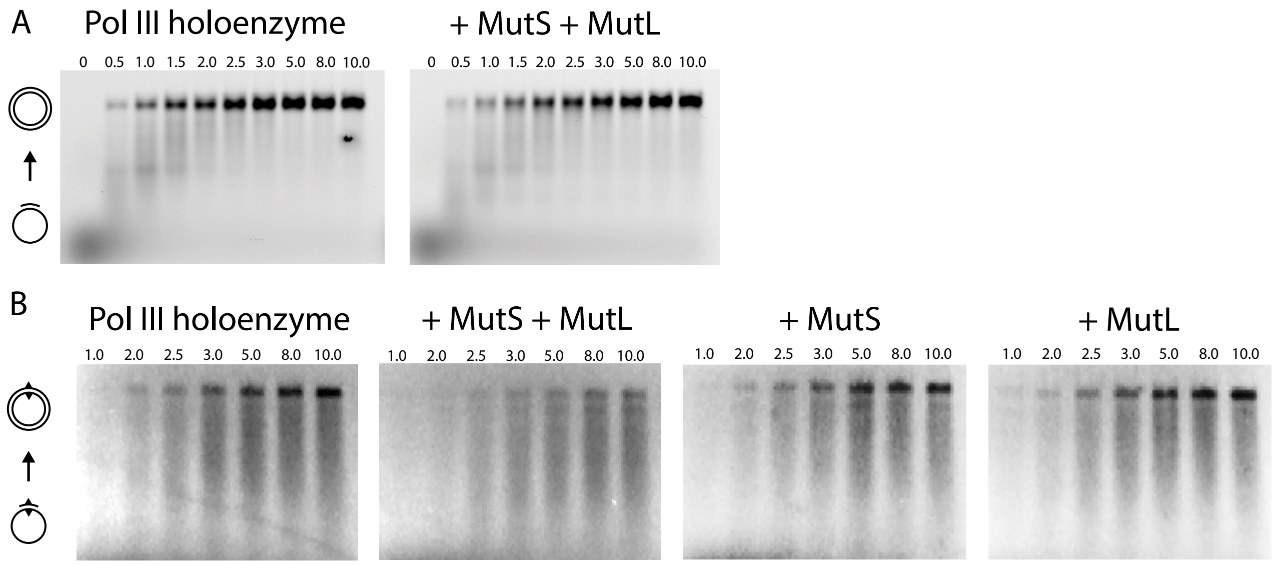


**Figure S1.** MutL is a mismatch and MutS-dependent inhibitor of DNA synthesis. (**A**) In the absence of mismatch, the presence of MutS and MutL does not inhibit DNA synthesis (**B**) Inhibition of DNA synthesis requires both MutS and MutL, as none of the enzymes alone can reduce the activity of the Pol III holoenzyme.
