# Supplementary material for: MutL binds to 3′ resected DNA ends and blocks DNA polymerase access
Source: Nucleic Acids Res. 2022 Jun 7;50(11):6224–34. doi: 10.1093/nar/gkac432 (PMC9226502; doi:10.1093/nar/gkac432)
Supplement: gkac432_Supplemental_Files [file gkac432_supplemental_files.zip › SupFig_S2_CryoEM_07feb.pdf]

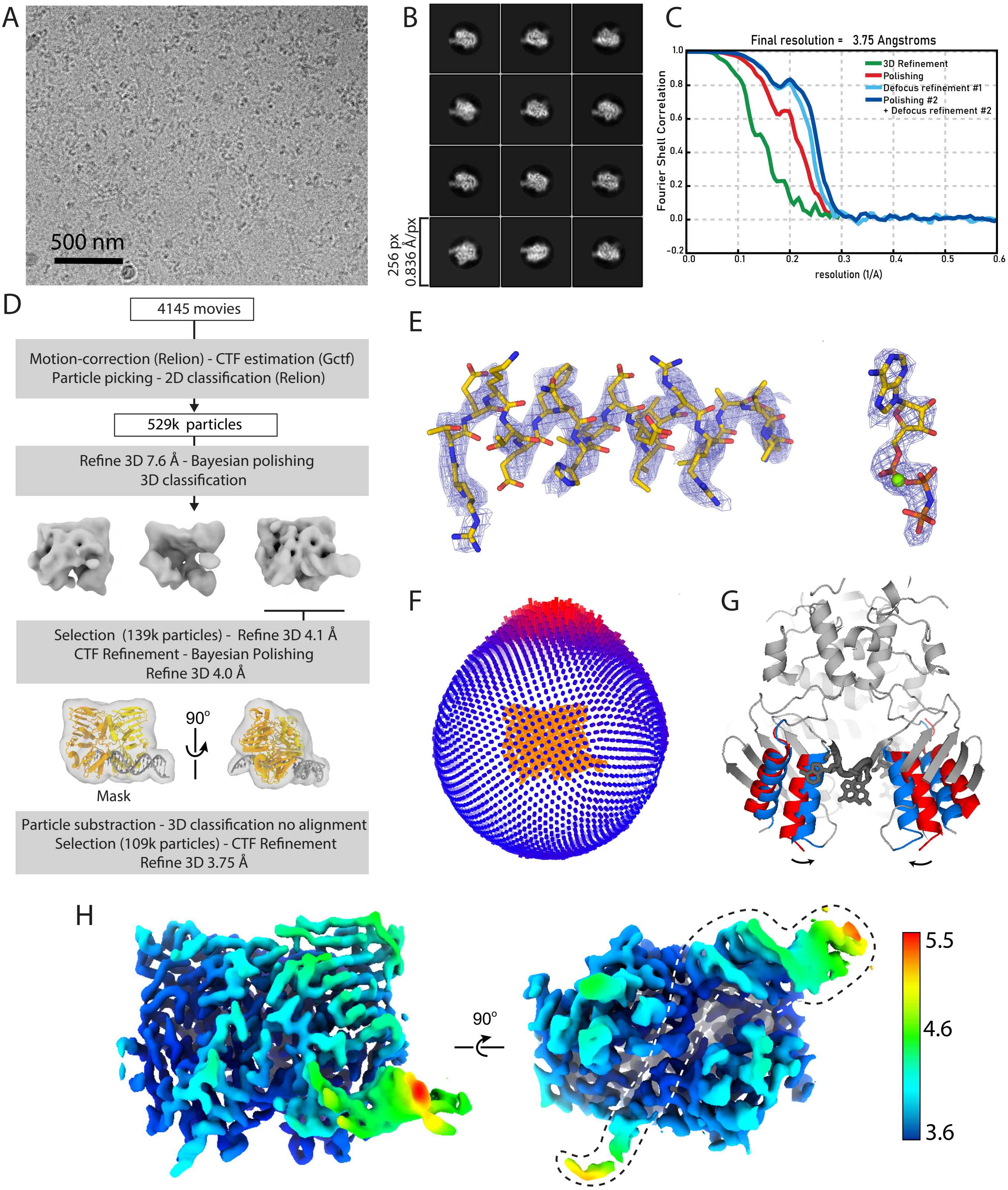

Figure S2. Cryo-EM structure of MutL bound to a 3' resected DNA end. (**A**) Representative micrograph. (**B**) Representative 2D class averages from the full dataset. (**C**) Fourier Shell Correlation between half-maps from subsequent refinements in the processing procedures. (**D**) Schematic representation of main data processing procedures. See methods for more details. (**E**) Detail of model fit to map. (**F**) Orientational distribution of the final set of particles. (**G**) Comparison between the crystal structure of MutL^{LN40} dimer in absence of DNA (Ban1999) and the cryo-EM structure presented in this work. The helices that move upon DNA binding (residues 265:281 & residues 313:331) are colored in red and blue for the DNA-free and DNA-bound structure, respectively. (**H**) Front and top view of the final map colored by local resolution. Dashed line represents the position of the DNA molecule.
