# Supplementary material for: MutL binds to 3′ resected DNA ends and blocks DNA polymerase access
Source: Nucleic Acids Res. 2022 Jun 7;50(11):6224–34. doi: 10.1093/nar/gkac432 (PMC9226502; doi:10.1093/nar/gkac432)
Supplement: gkac432_Supplemental_Files [file gkac432_supplemental_files.zip › SupFig_S3_Characterization_L_Mutants.pdf]

A

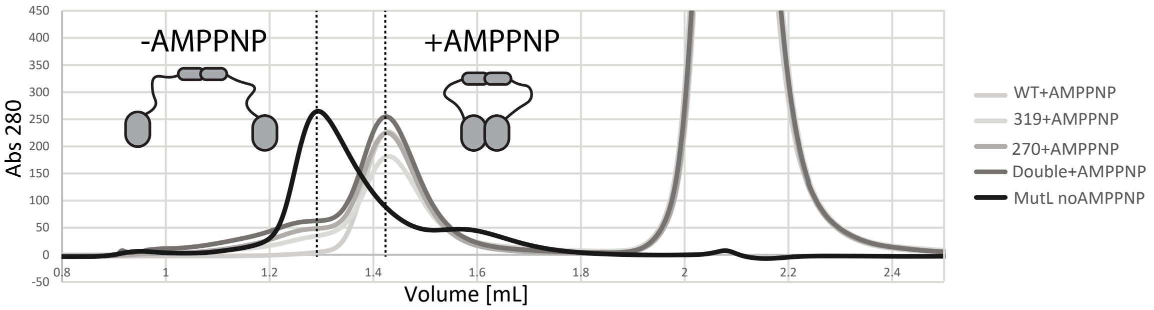

B

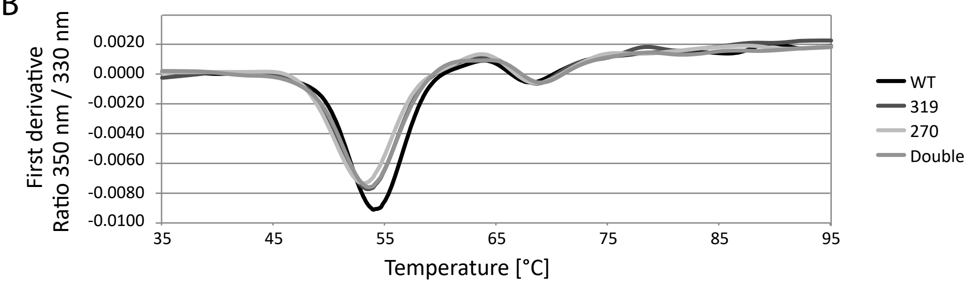

C

	T_1(°C)
WT	54.1
319	53.5
270	53.1
Double	53.6

Figure S3. Biochemical charachterization of MutL WT and mutants. (**A**) Size exclusion chromatography profiles in presence and absence of AMPPNP. In presence of AMPPNP the protein shifts to lower molecular weight because of the dimerization of the N-terminal domains, as indicated by the schematic representation of the MutL dimer. (**B**) Melting profiles of purified proteins. (**C**) Unfolding temperatures of MutL dimers.
